# CXCL4/CXCR3 axis regulates cardiac fibrosis by activating TGF‐β1/Smad2/3 signaling in mouse viral myocarditis

**DOI:** 10.1002/iid3.1237

**Published:** 2024-04-05

**Authors:** Jing Wei, Dan‐feng Wang, Cong‐cong Cui, Jia‐jia Tan, Ming‐yu Peng, Hong‐xiang Lu

**Affiliations:** ^1^ Department of Laboratory Medicine, Nanjing First Hospital Nanjing Medical University Nanjing China; ^2^ Department of Laboratory Medicine Jiangning Hospital Affiliated to Nanjing Medical University Nanjing China

**Keywords:** cardiac fibroblast, cardiac fibrosis, CXCL4, viral myocarditis

## Abstract

**Background:**

Severe myocarditis is often accompanied by cardiac fibrosis, but the underlying mechanism has not been fully elucidated. CXCL4 is a chemokine that has been reported to have pro‐inflammatory and profibrotic functions. The exact role of CXCL4 in cardiac fibrosis remains unclear.

**Methods:**

Viral myocarditis (VMC) models were induced by intraperitoneal injection of Coxsackie B Type 3 (CVB3). In vivo, CVB3 (100 TCID50) and CVB3‐AMG487 (CVB3: 100 TCID50; AMG487: 5 mg/kg) combination were administered in the VMC and VMC+AMG487 groups, respectively. Hematoxylin and eosin staining, severity score, Masson staining, and immunofluorescence staining were performed to measure myocardial morphology in VMC. Enzyme‐linked immunosorbent assay (ELISA) and quantitative reverse transcription polymerase chain reaction (qRT‐PCR) were performed to quantify inflammatory factors (IL‐1β, IL‐6, TNF‐α, and CXCL4). Aspartate aminotransferase (AST), lactate dehydrogenase (LDH), and creatine kinase‐myocardial band (CK‐MB) levels were analyzed by commercial kits. CXCL4, CXCR3B, α‐SMA, TGF‐β1, Collagen I, and Collagen III were determined by Western blot and immunofluorescence staining.

**Results:**

In vivo, CVB3‐AMG487 reduced cardiac injury, α‐SMA, Collagen I and Collagen III levels, and collagen deposition in VMC+AMG487 group. Additionally, compared with VMC group, VMC+AMG group decreased the levels of inflammatory factors (IL‐1β, IL‐6, and TNF‐α). In vitro, CXCL4/CXCR3B axis activation TGF‐β1/Smad2/3 pathway promote mice cardiac fibroblasts differentiation.

**Conclusion:**

CXCL4 acts as a profibrotic factor in TGF‐β1/Smad2/3 pathway‐induced cardiac fibroblast activation and ECM synthesis, and eventually progresses to cardiac fibrosis. Therefore, our findings revealed the role of CXCL4 in VMC and unveiled its underlying mechanism. CXCL4 appears to be a potential target for the treatment of VMC.

## Graphical abstract

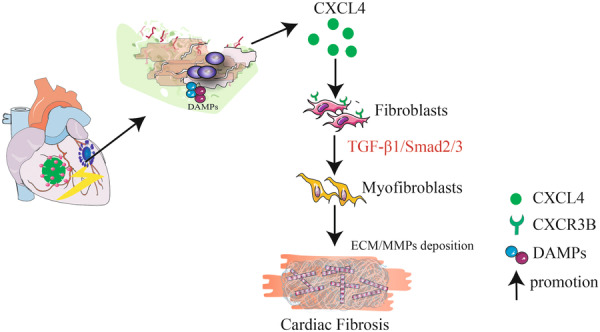

## 1 INTRODUCTION

Viral myocarditis (VMC) is the leading cause of sudden cardiac death in children and adolescents. Multiple viruses including enteroviruses, adenoviruses, and human herpes virus 6 have been associated with VMC.^1^, ^2^, ^3^ However, Coxsackievirus group B type 3 (CVB3), an enterovirus of the picornaviridae family, is known as the main pathogen of VMC.^4^ CVB3‐induced VMC is accompanied by early infiltration of immune cells and late cardiac fibrosis, which is due to the activation of MCFs leading to collagen production and abnormal accumulation of extracellular matrix.^5^

Cardiac fibrosis is a chronic progressive cardiovascular disease characterized by abnormal recruitment of immune cells in myocardial microenvironment, imbalance of epithelial–mesenchymal transition, accumulation of extracellular matrix, and irreversible scar formation.^6^ It also can increase left ventricular stiffness, disrupt cardiac conduction, and impair systolic and diastolic function.^7^ Cardiac fibrosis was independently associated with cardiovascular and all‐cause mortality. The activation of cardiac fibroblasts and the process of fibrosis are mediated by a variety of mechanisms. For example, after myocardial infarction (MI), TGF‐β1 (transforming growth factor‐β1) is increased near the infarct area, which can activate fibroblasts.^8^ Currently, no evidence‐based therapies have shown significant efficacy against fibrotic diseases, mainly because the mechanisms of cardiac fibrosis are not well understood.

CXC chemokine ligand 4 (CXCL4), also called platelet factor 4 (PF4), is a chemokine isolated from platelets. Unlike other chemokines, CXCL4 has a relatively weak chemo attractant role but extremely strong pro‐inflammatory function.^9^, ^10^ In addition, CXCL4 plays an important role in regulating cell apoptosis, survival, differentiation, proliferation, and migration.^11^, ^12^ Evidence has shown that CXCL4 was increased in inflammatory diseases, including atherosclerosis, inflammatory bowel disease, rheumatoid arthritis, and mesenteric ischemia/reperfusion injury.^13^, ^14^, ^15^ CXCR3 belongs to the CXC chemokine receptor family. It has two isoforms: CXCR3‐A and CXCR3‐B. CXCR3‐A and CXCR3‐B play opposing biological effects through distinct intracellular signals.^16^, ^17^ In general, activation of CXCR3‐A promotes cell growth, invasion, and survival, whereas CXCR3‐B appears to cause cell proliferation, inhibition, and apoptosis.^18^ However, CXCL4 binds specifically to CXCR3‐B.^19^ Data have shown that systemic sclerosis is a prototypic fibrotic disease in which CXCL4 is increased and strongly correlates with skin and lung fibrosis.^20^ Although this series of observations is suggestive of the potential profibrotic properties of CXCL4, it is currently unclear whether CXCL4 could play a direct role in initiating fibrotic processes via myofibroblast. Given the lack of effective therapies for fibrosis, this is a crucial void in information.

In the present study, we used a VMC model to investigate CXCL4 regulation of cardiac fibrosis. On Day 7, compared with control group, cardiac fibrosis degree in VMC group significantly increased since extracellular matrix (ECM) aggregation and profibrotic TGF‐β signaling were upregulated. However, it was obviously alleviated after blocking CXCL4 receptors. Our study establishes CXCL4 as a key component in fibrosis development and the potential of blocking CXCL4 as a promising therapeutic strategy.

## 2 MATERIAL AND METHODS

### 2.1 Mice

Male BALB/c (6–8 weeks) mice were obtained from Kavins Laboratory Animal Company. All animal experiments were performed in accordance with the guidelines for the care and use of Laboratory Animals (Ministry of Health, China, 1998).

### 2.2 CVB3 infection and AMG487 treatment

CVB3 virus (Nancy strain) was maintained through Hela cells (ATCC number: CCL‐2). Mice were intraperitoneally injected with CVB3 virus solution (100TCID50, 200 μL). AMG487 (MCE) was given to VMC mice by intraperitoneal (*i.p*.) injection every 2 days, 5 mg/kg. Seven days later, the model was successfully constructed, and the hearts and serum of the mice were collected for the experiment.

### 2.3 Cell culture

MCF were purchased from BeNa Culture Collection and were cultured with Dulbecco's modified eagle medium (Gibco) containing 10% fetal bovine serum in a 5% CO_2_ incubator. The 3–6 passages of cells were used for experiments. MCF were stimulated with CXCL4 (5 μg/mL, PeproTech) for 24 h. The 1 μM AMG487 or 10 μM LY294002 (MCE) was added for 1 h before the stimulation with CXCL4.

### 2.4 Quantitative reverse transcription polymerase chain reaction (RT‐PCR)

An RNA extraction kit (Beyotime) was used to extract total RNA from cells and tissues. Purified RNA was reverse‐transcribed into cDNA, then amplified by SYBR‐Green master mix kit. The relative expression of genes was calculated by the 2^−ΔΔ*C*^t method. Primer sequences are shown in Table 1.

**Table 1:** Primer sequences.

Target DNA	Forward primer (5′ → 3′)	Reverse primer (5′ → 3′)
Collagen‐I	TTGCTTCCCAGATGTCCTATG	CTTCCCCATCATCTCCATTCT
Collagen‐III	CCAGACACTCATGTTGCCTGTTC	GAGGCTCCGGTTGGTGCTTA
α‐SMA	GTCCCAGACATCAGGGAGTAA	TCGGATACTTCAGCGTCAGGA
β‐actin	GAAGTCCCTCACCCTCCCAA	GGCATGGACGCGACCA

### 2.5 Western blot

Radioimmunoprecipitation assay buffer was used for preparing whole cell lysates. Protein was separated by sodium dodecyl‐sulfate polyacrylamide gel electrophoresis and transferred to polyvinylidene fluoride membranes (Millipore). In addition, 1% bovine serum albumin (BSA) was added to block the membranes. Then, the membranes were washed three times with TBS‐0.1% Tween 20 (TBST). The washed membranes were incubated with primary antibodies at 4°C overnight. The following primary antibodies were used: anti‐GAPDH (1:30,000, Cat: 60004‐1‐Ig; Proteintech), Collagen‐I (1:1000, Cat: 14695‐1‐AP; Immunoway), Collagen‐III (1:500, Cat: 22734‐1‐AP; Proteintech), α‐smooth muscle actin (α‐SMA) (1:3000, Cat: 14395‐1‐AP; Proteintech), anti‐Actin (1:5000, Cat: BS6007M; Bioworld), anti‐CXCL4 (1:3000; Proteintech), anti‐TGF‐β1 (1:1000, Cat: CPA2154; Cohesion), and anti‐p‐Smad2/3 (1:1000, Cat: BS1838; Bioworld). The membranes were then incubated with the following horseradish peroxidase‐conjugated secondary antibodies: Afterwards, the secondary antibody (1:10,000; Proteintech) was incubated at room temperature for 1 h. Finally, the antigen‐antibody reactions were visualized by chemiluminescence (ECL) kit, and the intensity of protein bands was quantified by using ImageJ software.

### 2.6 Enzyme‐linked immunosorbent assay (ELISA)

The levels of CXCL4, IL‐1β, IL‐6, TNF‐α, and TGF‐β1 (Lianke) were quantified in serum or culture media by a quantitative sandwich enzyme immunoassay according to the manufacturer's instructions.

### 2.7 Immunofluorescence staining

The mice were killed by neck breaking, and the middle heart tissue was selected to make paraffin sections. Then, the prepared paraffin sections were placed in oven at 60°C and waited for 1 h. The slices were then soaked with xylene for two times, about 15 min each time. After that, the slices were immersed in gradient anhydrous ethanol (100%, 95%, 85%, 70%) for one time. 6 min each time. Subsequently, boil the slices in citrate buffer for 10 min. Then, take out and cool for about 30 min naturally. Weigh 0.05 g BSA and add it into 1 mL phosphate‐buffered saline (PBS) solution to prepare 5% BSA, then drip it into the tissue on the section and wait for 1 h. Sections were stained with the primary antibody overnight at 4°C. The primary antibody was discarded and washed with PBS for three times, for about 10 min each time. Secondary antibody (1:200) was added and incubated for 1 h at room temperature and away from light. The secondary antibody was recovered and cleaned with PBS for three times, 15 min each time. Diluted DAPI (4′,6‐diamidino‐2‐phenylindole) was added and incubated in the dark for about 15 min. Then, the slices were cleaned with PBS for three times, 15 min each time.

### 2.8 Histopathological examination of the heart

The heart tissues were fixed in 4% polyformaldehyde and embedded in paraffin. Then sections were stained with hematoxylin–eosin (H&E). H&E staining was used to analyze the level of inflammation under a microscope in random order.

### 2.9 Masson staining

Mouse heart tissue was fixed in 4% paraformaldehyde overnight. Samples were embedded in paraffin, and 7 µm sections were obtained for staining. The standard procedures for the Masson's staining were performed.

### 2.10 Immunohistochemistry (IHC)

Heart tissue was fixed in 4% paraformaldehyde, embedded in paraffin, and analyzed by immunohistochemical analysis. Heart tissue was incubated with anti‐CXCL4 (1:100; Proteintech) at 4°C overnight. Then, heart tissue was incubated with biotin‐labeled secondary antibodies. The immunoreaction signal was developed with DAB (3, 3′‐diaminobenzidine) staining. Finally, sections were viewed under a light microscope.

### 2.11 Examination of myocardial markers

The levels of lactate dehydrogenase (LDH), creatine kinase‐myocardial band (CK‐MB), and aspartate aminotransferase (AST) in serum were measured using detection kit (MULTI Sciences).

### 2.12 Statistical analysis

Statistical analysis was performed using GraphPad Prism 8.0 (GraphPad Software, Inc.). Data are presented as mean ± standard deviation. A *t* test was used to compare the data between two groups and the differences between multiple groups were analyzed via a one‐way analysis of variance. A value of *p < .05 was considered as statistically significant.

## 3 RESULTS

### 3.1 Expression of CXCL4 increases during viral myocarditis

**Figure 1:**
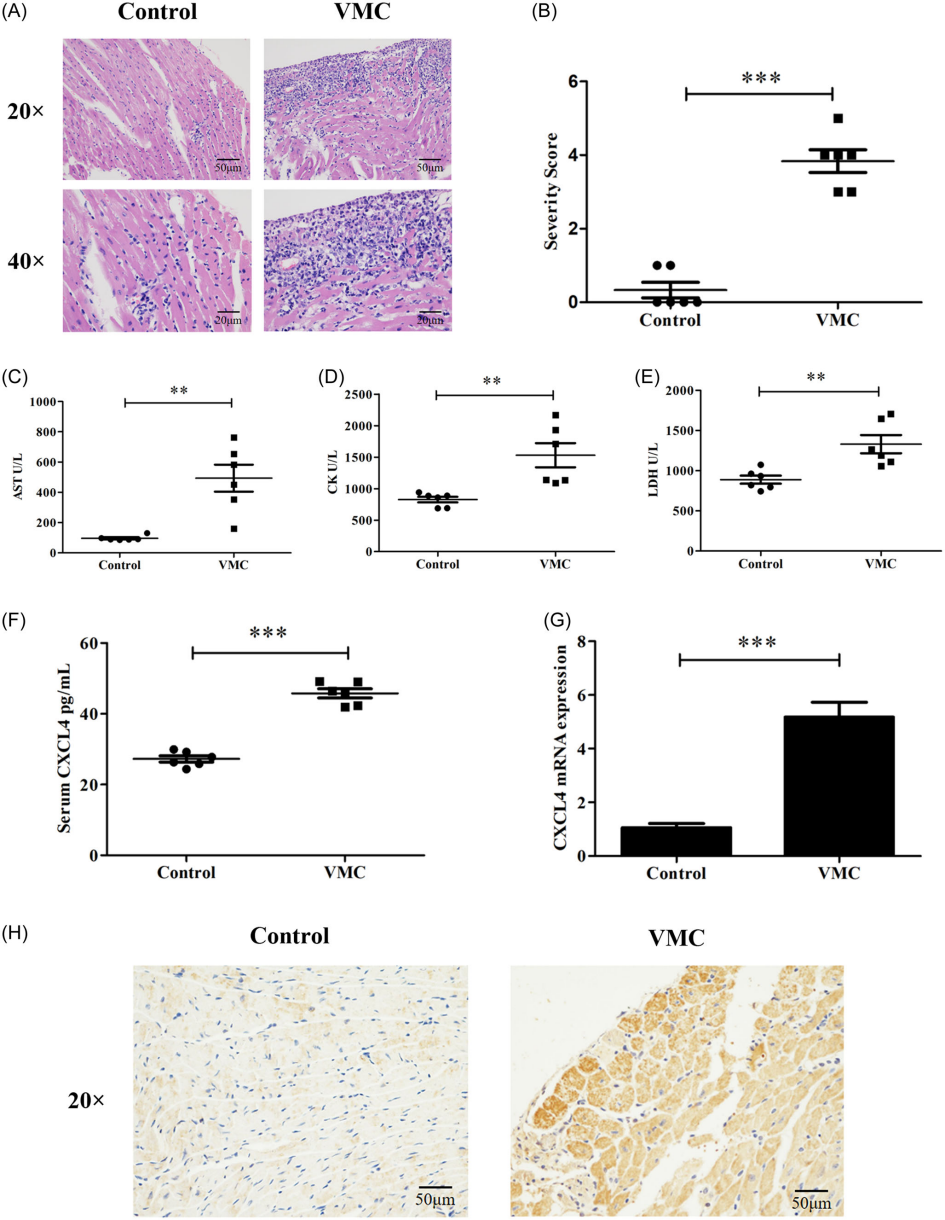
Expression of CXCL4 increases during viral myocarditis. (A) Pathological changes in the heart tissue assessed using H&E staining. (B) Inflammation score. (C)–(E) Levels of serum CK, AST, and LDH in mice. (F) and (G) Level of CXCL4 in the serum (F) and messenger RNA expression of CXCL4 in the heart tissues (G) were measured using ELISA and qPCR, respectively. (H) Images of immunofluorescence staining showing the expression of CXCL4 in heart sections (*n* = 6). Data are presented as mean ± standard deviation. AST, aspartate aminotransferase; CK, creatine kinase; CXCL4, CXC chemokine ligand 4; ELISA, enzyme‐linked immunosorbent assay; H&E, hematoxylin and eosin; LDH, lactate dehydrogenase; qPCR, quantitative polymerase chain reaction; VMC, viral myocarditis. *p < .05, **p < .01, ***p < .001.

To investigate the relationship between viral myocarditis and CXCL4, we first generated the acute viral myocarditis model by intraperitoneal injection of 200 μL TCID50 of CVB3 into 6‐week‐old male Balb/c mice. Mice were killed on Day 7 postinfection. HE staining and pathological score showed significant infiltration of inflammatory cell in the myocardium (Figure 1A,B). In addition, the sensitive indicators of myocardial injury serum CK‐MB, lactate dehydrogenase, and aspartate aminotransferase were higher in VMC group than that in control group (Figure 1C–E). All results indicate that the model of VMC was successfully constructed. Since the fact that CXCL4 is a protein with pro‐inflammatory and fibrotic functions, which is expressed in various validated diseases and participates in disease progression, we investigated the relationship between CXCL4 and VMC. ELISA was used to detect the level of CXCL4 in serum. The results showed that compared to the control group, the serum CXCL4 levels in the VMC group increased (Figure 1F). In addition, the result of RT‐PCR showed that gene expression of CXCL4 in VMC group was upregulated at the transcript level (Figure 1G). Consistent with the above results, immunohistochemical staining showed that expression of CXCL4 is higher in the heart of the VMC group than control group (Figure 1H). Together, these results suggest that expression of CXCL4 increases during viral myocarditis.

### 3.2 CXCL4 promotes cardiac inflammatory damage

**Figure 2:**
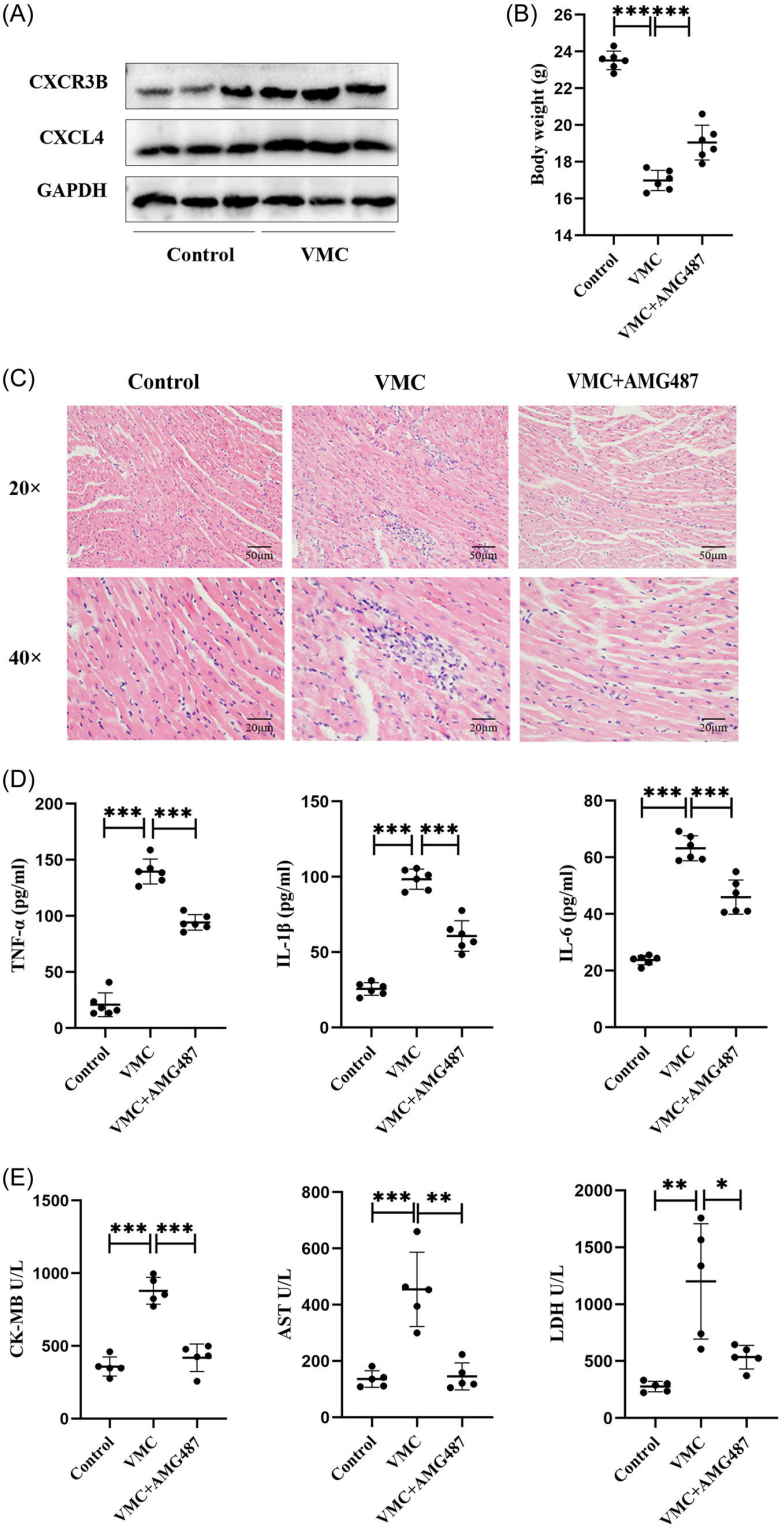
CXCL4 promotes cardiac inflammatory damage. (A) Western blot analysis images of CXCR3B and CXCL4 levels. (B) Body weight of mice. (C) Pathological changes in the heart tissue assessed using H&E staining. (D) Level of TNF‐α, IL‐1β, and IL‐6 in the serum were measured using ELISA. (E) Level of serum CK‐MB, AST, and LDH in mice (*n* = 6). Data are presented as mean ± standard deviation. AST, aspartate aminotransferase; CK‐MB, creatine kinase‐myocardial band; CXCL4, CXC chemokine ligand 4; ELISA, enzyme‐linked immunosorbent assay; H&E, hematoxylin and eosin; IL‐1β, interleukin‐1β; IL‐6, interleukin‐6; LDH, lactate dehydrogenase; TNF‐α, tumor necrosis factor‐α; VMC, viral myocarditis. *p < .05, **p < .01, ***p < .001.

To investigate whether CXCL4 plays a role in VMC, Western blot was used to detect protein expression of CXCL4 and CXCR3B. The results showed that the proteins expression of CXCL4 and CXCR3B was significantly increased in the hearts of VMC mice (Figure 2A). CXCL4 may participate in the pathogenesis and progression of VMC by binding to its receptors. VMC can cause inflammatory damage to the heart. Therefore, we investigated the effect of CXCL4 on cardiac inflammatory damage. AMG487 (CXCL4 receptor antagonist) was intraperitoneally injected into VMC mice. On the seventh day, the body weight of mice was measured and it was found that the VMC mice had a significant decrease in body weight, while the VMC+AMG487 group had a relatively higher body weight (Figure 2B). The results of HE staining showed that compared with the VMC group, the infiltration of inflammatory cells in the heart of the VMC+AMG487 group mice was significantly reduced (Figure 2C). The levels of inflammatory cytokines TNF‐α, IL‐1β, and IL‐6 in mouse serum were detected through ELISA. Compared with the VMC group, the levels of inflammatory cytokines TNF‐α, IL‐1β, and IL‐6 in the VMC+AMG487 group mice significantly reduced (Figure 2D). In addition, the myocardial zymogram is also used to detect cardiac injury. The results showed that AMG487 treatment significantly reduced serum levels of CK‐MB, AST, and LDH (Figure 2E). The above results indicate that CXCL4 promotes cardiac inflammatory damage.

### 3.3 CXCL4 promotes the differentiation of cardiac fibroblasts into myofibroblasts

**Figure 3:**
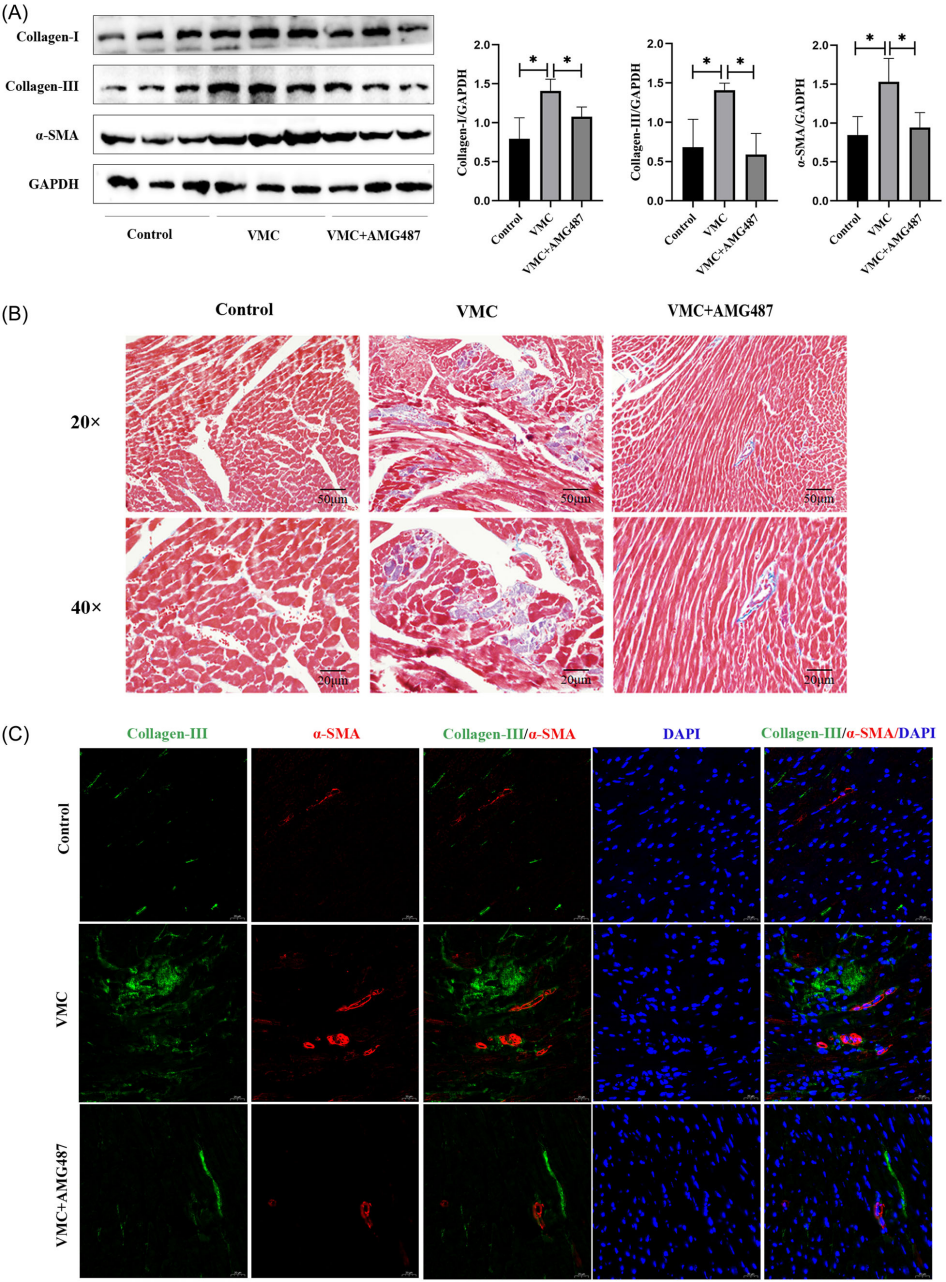
CXCL4 promotes the differentiation of cardiac fibrosis into myofibroblasts. (A) Western blot analysis images of Collagen‐I, Collagen‐III, and α‐SMA levels and protein grayscale analysis. (B) Collagen deposition in the heart tissue determined by Masson staining. (C) Images of immunofluorescence staining showing the expression of Collagen‐III and α‐SMA in heart sections (*n* = 6). Data are presented as mean ± standard deviation. CXCL4, CXC chemokine ligand 4; SMA, smooth muscle actin; VMC, viral myocarditis. *p < .05, **p < .01, ***p < .001.

VMC can cause cardiac fibrosis. Therefore, we investigated the effect of CXCL4 on cardiac fibrosis. Western Blot is used to detect protein expression in mouse heart tissue. Compared with the VMC group, the protein expression of Collagen I, Collagen III, and SMA in the VMC+AMG487 group was significantly reduced (Figure 3A). The results of Masson staining showed that compared with the VMC group, the deposition of collagen in the heart of the VMC+AMG487 group mice was significantly reduced (Figure 3B). In addition, we also detected the expression of Collagen III and α‐SMA in cardiac tissue through immunofluorescence staining. The results showed that compared with the VMC group, the expression of Collagen III and α‐SMA in the VMC+AMG487 group significantly reduced (Figure 3C). The above results indicate that CXCL4 promotes cardiac fibrosis.

### 3.4 CXCL4 mediates cardiac fibrosis by activating the TGF‐β1/Smad2/3 signaling pathway

**Figure 4:**
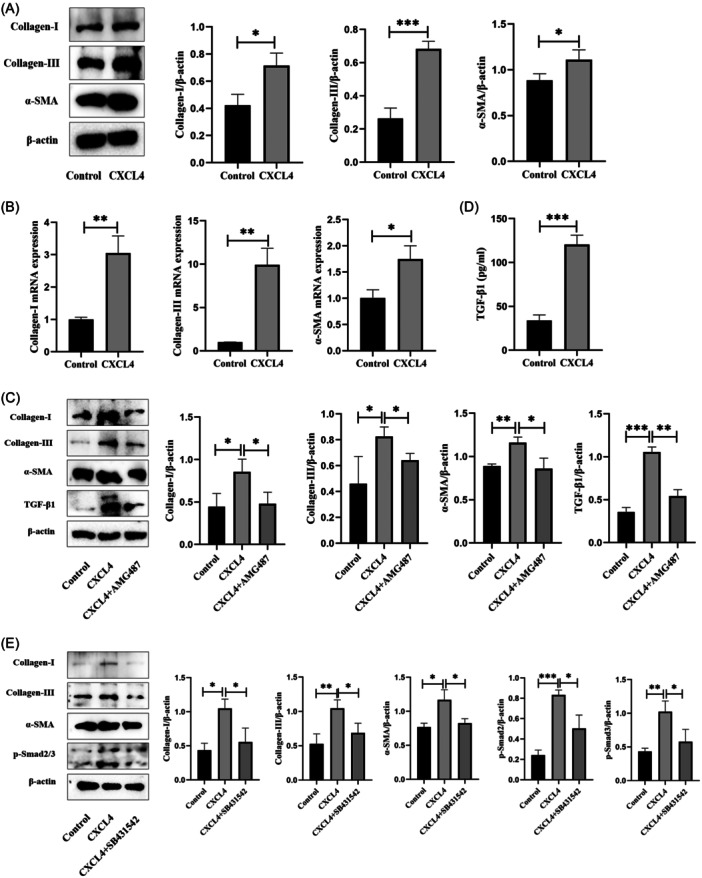
CXCL4 mediates cardiac fibrosis by activating the TGF‐β1/Smad2/3 signaling pathway. (A) Western blot analysis images of Collagen‐I, Collagen‐III, and α‐SMA levels and protein grayscale analysis. (B) messenger RNA expression of Collagen‐I, Collagen‐III, and α‐SMA were measured using qPCR. (C) Western blot analysis images of Collagen I, Collagen III, α‐SMA, and TGF‐β1 levels and protein grayscale analysis. (D) Level of TGF‐β1 in the cell supernatant was measured using ELISA. (E) Western blot analysis images of Collagen I, Collagen III, α‐SMA, and p‐Smad2/3 levels and protein grayscale analysis (*n* = 6). Data are presented as mean ± standard deviation. CXCL4, CXC chemokine ligand 4; ELISA, enzyme‐linked immunosorbent assay; qPCR, quantitative polymerase chain reaction; SMA, smooth muscle actin; TGF, transforming growth factor. *p < .05, **p < .01, ***p < .001.

To further verify that CXCL4 promotes cardiac fibrosis, MCF was treated with CXCL4 in vitro. The protein expression of Collagen I, Collagen III, and α‐SMA was detected through Western blot (Figure 4A). The results indicate that CXCL4 treatment can activate MCF. Similarly, results of qRT‐PCR showed that CXCL4 can promote gene expression of Collagen I, Collagen III, and α‐SMA in MCF, indicating that CXCL4 can promote MCF activation (Figure 4B). In addition, we also found that pretreatment of AMG487 can inhibit CXCL4‐mediated MCF activation through Western blot (Figure 4C). These results indicate that CXCL4 mediates MCF activation by binding to its receptor. Next, we investigated the mechanism of CXCL4‐induced MCF activation. So far, the most widely studied mediator for promoting fibroblast activation is the TGF‐β growth factor family. TGF‐β1 plays a crucial role in regulating fibrosis in various organs, so detecting the content of TGF‐β1 in serum can be used as a tool for diagnosing fibrosis. In the TGF‐β growth factor family, TGF‐β1 is the most important factor involved in inducing pathological fibrosis. Therefore, we detected the level of TGF‐β1 in the cell supernatant by ELISA after treating MCF with CXCL4. The results showed a significant increase in TGF‐β1 levels in the supernatant of the CXCL4 group compared to the control group (Figure 4D). Consistent with the above results, the results of Western Blot showed that AMG487 treatment can inhibit the protein expression of Collagen I, Collagen III, α‐SMA, and TGF‐β1 (Figure 4E). During the classic pathway of TGF‐β signaling, Smad2/3 phosphorylation occurs, followed by binding to Smad4 and cotranslocation to the nucleus. Finally, this complex acts as a transcription factor, inducing the activation of profibrotic genes, leading to fibrosis. To verify whether CXCL4 mediates MCF activation through the activation of TGF‐β1/Smad2/3 signaling pathway, SB431542 (TGF‐β receptor kinase inhibitor) was used to preprocess MCF. The results of Western Blot showed that pretreatment of SB431532 significantly inhibited the protein expression of Collagen I, Collagen III, α‐SMA, and p‐Smad2/3 (Figure 4F). In summary, the above results indicate that CXCL4 mediates cardiac fibrosis by activating the TGF‐β1/Smad2/3 signaling pathway.

## 4 DISCUSSION

In this study, we demonstrated that VMC promotes the production of CXCL4. Furthermore, We also confirmed that CXCL4 is involved in the cardiac fibrosis by activating the TGF‐β1/Smad2/3 pathway.

VMC is an inflammatory disease of the heart caused by viral infection, which is a significant cause of dilated cardiomyopathy (DCM).^21^ About one‐fifth of the patients with VMC will progress to dilated cardiomyopathy, leading to heart failure or sudden death. VMC can lead to damage of cells, infiltration of immune cells, and activation of cardiac fibroblasts, subsequently leading to cardiac fibrosis.^22^, ^23^, ^24^ However, the precise molecular mechanisms underlying the association between VMC and cardiac fibrosis are still unclear and need to be investigated further. VMC can cause infiltration of inflammatory cells, activation of cardiac fibroblasts, deposition of collagen, and ultimately lead to cardiac fibrosis as the disease progresses.^25^ Excessive cardiac fibrosis is a major problem in almost all types of cardiovascular diseases, mainly attributed to activation and excessive proliferation of CF.^26^ Activated CF, known as myofibroblasts (MFB), can produce a large amount of ECM, including collagen, matrix metalloproteinases, and so on.^27^ Compared to CF, MFB expresses high levels of α‐SMA.^28^ Inflammatory stimulation and myocardial cell death are usually the initial factors for excessive ECM secretion by fibroblasts.^29^ When the heart is damaged, myocardial cells die and secrete various cytokines, which migrate CF to the injured site followed by CF proliferation and activation into MFB. MFB secretes ECM such as collagen, ultimately forming scar tissue mainly composed of collagen fibers to complete the repair of heart tissue.^30^ Moderate collagen synthesis has a beneficial effect on heart repair, but excessive collagen deposition can have adverse effects on the heart.^31^ Multiple studies have shown that VMC‐induced cardiac inflammatory damage can lead to significant release of cytokines. These cytokines can exert fibrotic effects, such as TGF‐β, AngII, and so on.^32^, ^33^ In this study, we demonstrated that VMC promotes the production of CXCL4 and the expression of CXCR3B.

CXCL4 is a member of the CXC chemokine family, released from activated platelets.^34^, ^35^ CXCL4 has multiple functions, such as antiangiogenic, pro‐inflammatory, and fibrogenic.^36^ Studies have shown that CXCL4 deficiency in the hematopoietic system can inhibit fibrosis in myeloproliferative tumors. CXCL4 knockout can alleviate cardiac fibrosis in a fibrotic model caused by transverse aortic contraction.^37^ Our study found that CXCL4 treatment promotes MCF activation and AMG487 treatment alleviates cardiac fibrosis in VMC mice, which indicates that CXCL4 promotes cardiac fibrosis in VMC mice. TGF‐β1/Smad2/3 signaling pathway is a classic pathway that induces fibrosis. To verify whether CXCL4 induces MCF activation by activating the TGF‐β1/Smad2/3 signaling pathway, pretreatment was performed with AMG487 and SB431532. It was found that AMG487 and SB431542 inhibited the profibrotic effect of CXCL4. Together, we found that CXCL4 promotes cardiac fibrosis in VMC mice and relies on the activation of the TGF‐β1/Smad2/3 signaling pathway.

**Figure 5:**
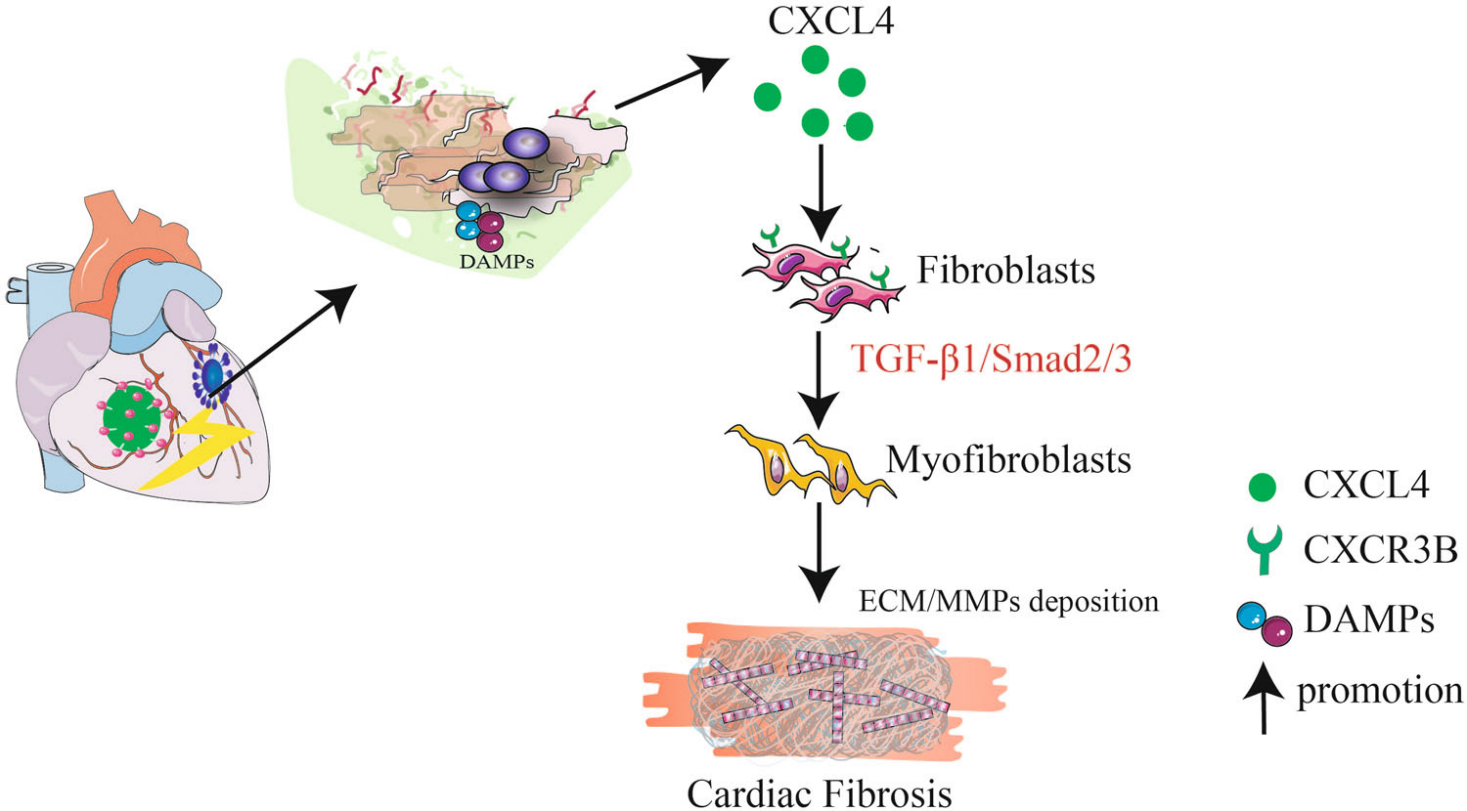
Working hypothesis. The diagram shows the working hypothesis based on the present work and the previously established mechanisms to upregulate CXCL4 expression in the development of cardiac fibrosis. CXCL4, CXC chemokine ligand 4; ECM, extracellular matrix; MMPs, matrix metalloproteinases.

In conclusion, our results suggest the vital role of CXCL4 in the development of viral myocarditis. Reducing CXCL4 production can alleviate cardiac fibrosis. In addition, CXCL4 promotes cardiac fibrosis through activating the TGF‐β1/Smad2/3 signaling pathway. Inhibition of CXCL4 secretion may be considered a potential therapeutic target for myocarditis treatment (Figure 5).

## AUTHOR CONTRIBUTIONS

**Jing Wei**: Formal analysis; investigation; writing—original draft. **Dan‐feng Wang**: Formal analysis; software. **Cong‐cong Cui**: Formal analysis; software. **Jia‐jia Tan**: Investigation; writing—original draft. **Ming‐yu Peng**: Data curation; software. **Hong‐xiang Lu**: Conceived and designed the study.

## CONFLICT OF INTEREST STATEMENT

The authors declare no conflict of interest.

## Supporting information

Supporting information.

## Data Availability

The data that support the findings of this study are available from the corresponding author upon reasonable request.
